# Mechanisms of Mitochondria-Mediated Apoptosis During *Eimeria tenella* Infection

**DOI:** 10.3390/ani15040577

**Published:** 2025-02-17

**Authors:** Rui Bai, Hui Wang, Tiantian Yang, Yuqi Yan, Shuying Zhu, Chenyang Lv, Yang Pei, Jiale Guo, Jianhui Li, Xiaozhen Cui, Xiaoling Lv, Mingxue Zheng

**Affiliations:** 1 College of Veterinary Medicine, Shanxi Agricultural University, Taigu, Jinzhong 030801, China; 2 Shanxi Key Laboratory for Modernization of TCVM, College of Veterinary Medicine, Shanxi Agricultural University, Taigu, Jinzhong 030801, China; 3 College of Animal Science, Shanxi Agricultural University, Taigu, Jinzhong 030801, China

**Keywords:** *Eimeria tenella*, apoptosis, mitochondrial apoptosis, cytochrome c, cyclosporine A, TMPD

## Abstract

Coccidiosis in chickens is a parasitic disease caused by *Eimeria* species, resulting in significant economic losses to the poultry industry. Among these species, *Eimeria tenella* is considered the most virulent pathogen, with its infection strongly associated with the apoptotic response of host cells. *Eimeria tenella* modulates host cell apoptosis in a stage-specific manner, suppressing apoptosis in the early phase to promote its intracellular development and triggering apoptosis in later stages to facilitate parasite egress and disease progression. This study established an in vitro infection model using 60 fifteen-day-old chick embryo cecal epithelial cells and infecting the cells with *Eimeria tenella* sporozoites at a 1:1 ratio of host cells to sporozoites. The aim was to examine the relationship between parasitic infection and the apoptotic response of host cells in the chick embryo cecal epithelial cells infected with *E. tenella*. The roles of the mitochondrial permeability transition pore (MPTP) and cytochrome c in intrinsic apoptosis were examined through the application of cyclosporine A (CsA), N, N, N’, N’-tetramethyl-1,4-phenylenediamine (TMPD), and ascorbate (Asc). TUNEL staining, ELISA, and flow cytometry were performed to evaluate apoptotic rates. CsA, TMPD, and Asc significantly (*p* < 0.01) decreased cytochrome c release, caspase-9 activation, and apoptotic rates from 24 to 120 h post-*E. tenella* infection. These findings highlight the significance of cytochrome c-mediated, mitochondria-dependent apoptotic pathways in parasitized chick embryo cecal epithelial cells.

## Simple Summary

Coccidiosis is a disease caused by various *Eimeria* species that parasitize the intestinal tract of chickens. *Eimeria* infections cause significant economic losses in the poultry industry due to reduced weight gain, increased feed conversion ratios, and high mortality rates. Among the known *Eimeria* species, *Eimeria tenella* is considered the most pathogenic, although its prevalence can vary across different regions. *Eimeria tenella* induces apoptosis in host cells. This study systematically investigated the mechanisms by which *E. tenella* induces host cell apoptosis via the mitochondrial permeability transition pore (MPTP) and cytochrome c. Using inhibitors, this study demonstrated that the inhibition of mitochondrial pathways significantly reduces host cell apoptosis following infection. Unraveling the mechanisms of *Eimeria tenella*-induced apoptosis can contribute to the development of targeted therapies, novel vaccines, and enhanced disease control measures to effectively control infection and mitigate economic losses.

## 1. Introduction

Coccidiosis in chickens is a parasitic disease caused by *Eimeria* spp. infecting the intestinal epithelial cells of chickens, which poses a severe threat to chicken health and imposes substantial economic losses on the global poultry industry [1,2,3]. *Eimeria*’s pronounced host specificity and site specificity in chickens significantly contribute to severe infections [4]. The infection of chickens by *Eimeria* starts when sporulated oocysts are ingested from the environment (such as feces or contaminated litter), and the released sporozoites invade the intestinal epithelial cells [5]. The infection disrupts the host’s mucosal cells, enhances cellular permeability, and exudes nutrients and proteins [6]. It also interferes with the digestion and absorption of proteins and other nutrients [7], collectively contributing to the subclinical and clinical symptoms of coccidiosis, including depression, disheveled feathers, reduced appetite, bloody stools, and potentially death [8]. The economic burden of chicken coccidiosis and its control has risen from USD 800 million annually in 2002 to USD 3 billion in 2006 and GBP 10.4 billion (about USD 12.91 billion) in 2020 [9,10,11]. These costs encompass direct losses from chicken mortality and indirect losses such as diminished broiler weight gain, higher feed conversion rates, and lower egg production [11].

Currently, various strategies are employed to prevent and control chicken coccidiosis. Anticoccidial chemical agents, including synthetic coccidiostats and ionophore coccidiostats [12], have long been the dominant strategy in modern poultry production. However, issues such as drug resistance and residues highlight the necessity of finding alternative antibiotic approaches [13]. Vaccines, which elicit immune responses to provide protection [14], include live virulent, attenuated, and genetically engineered types [6]. However, immunization failure causing coccidiosis and the high costs associated with vaccines have constrained their broader application [6]. Extensive research has been conducted on alternative therapies, including prebiotics, probiotics, plant and fungal extracts, and essential oils [15]. These natural compounds primarily function by modulating gut microbiota and immune responses and may not directly target parasites, underscoring the necessity of elucidating their toxicological characteristics and mechanisms of action [16,17].

*Eimeria tenella*, which lives in chickens’ cecum, is recognized as one of the most pathogenic chicken coccidia discovered so far [18,19,20]. *E. tenella* infection of cecal epithelial cells leads to apoptosis, a critical stage in disease pathogenesis [21]. Understanding the mechanisms underlying *E. tenella*-induced apoptosis may offer new avenues for therapeutic intervention. Controlling apoptosis is crucial for managing infections, as it helps prevent excessive parasite replication, modulate immune responses, and limit disease progression, thereby improving therapeutic outcomes.

To expand upon these insights, a key component of the mitochondrial apoptosis pathway is cytochrome c, which plays a central role in initiating the apoptotic cascade [22,23,24]. A loss of mitochondrial membrane potential leads to increased cytochrome c release, which then translocates to the cytoplasm and forms the apoptosome by oligomerizing with procaspase-9 and Apaf-1 [25]. Caspase-9 activates caspase-3 to mediate the final stages of apoptosis [26,27,28]. This pathway has been extensively investigated in mammalian cells, and relevant studies have also been conducted in parasitic infections. *Toxoplasma gondii* inhibited mitochondrial apoptotic pathways by targeting cytochrome c and caspase-9 [29]. *Leishmania* elevates cytoplasmic cytochrome c levels and inhibits apoptosis in infected macrophages [30]. These observations indicate that mitochondrial pathways play an essential role in mediating the apoptotic response of host cells to parasitic infections. The regulation of apoptosis is complex and involves numerous cellular components. However, the mechanisms governing cytochrome c release remain controversial. Mitochondrial permeability transition pore (MPTP) opening occurs in response to *E. tenella* infection [31]. This study dissected the mechanisms by which *E. tenella* induces apoptosis in chicken embryonic cecal epithelial cells, emphasizing the role of MPTP opening and cytochrome c release in intrinsic apoptosis.

## 2. Materials and Methods

### 2.1. Parasites

Virulent *E. tenella* Shanxi strains (EtSX01) were obtained from the Laboratory of Veterinary Pathology, Shanxi Agricultural University. A total of 20 specific pathogen-free (SPF) chicks (20 days old) were infected orally with *E. tenella* (5000 sporulated oocysts). Oocysts were isolated from fecal samples collected 7–8 days post-infection using standard salt flotation. The specific procedure was as follows: After removing debris and weighing, fecal samples were mixed with ten times their weight of cooled, boiled water and glass beads, shaken for 20 min to facilitate oocyst release, and then sieved. The filtrate was then centrifuged and washed three times, followed by flotation centrifugation using ten times its volume of saturated saline. The oocyst layer in the supernatant was collected, with flotation repeated if necessary. Finally, the oocyst layer was diluted sevenfold with cooled, boiled water, centrifuged, and the purified oocysts were collected [32,33,34]. Oocysts were cultured at 28 °C in 2.5% potassium dichromate to achieve ≥ 90% sporulation. Sporocysts were mechanically released using a glass homogenizer until ≥80% excystation was reached. Released sporocysts were digested for 1 h at 41 °C in 0.75% (*v*/*v*) trypsin (Solarbio, Beijing, China) combined with 10% bile from SPF chicks. Samples were then centrifuged and filtered through a 1400 mesh sieve. Free sporozoites were then concentrated to 1.5 × 10^5^/mL in a medium composed of low-glucose DMEM (HyClone, Logan, UT, USA), MEM199 (Hyclone) (MEM199: DMEM = 2:1 *v*/*v*), 1.5% (*v*/*v*) FBS (Sijiqing, Hangzhou, China), and the following supplements: EGF (PeproTech, Rocky Hill, NJ, USA) (0.02 μg/mL), glucose (Tianjin Chemical Agent Corp, Tianjin, China) (0.3 mg/mL), penicillin (100 U/mL), streptomycin (100 U/mL), heparin (0.1 mg/mL), sodium pyruvate (1.1 mg/mL), insulin (0.05 μg/mL), L-glutamine (0.5 mM), folic acid (0.05 μg/mL), vitamin B6 (0.05 μg/mL), and vitamin B1 (0.05 μg/mL) (all from Solarbio, Beijing, China). TMPD was purchased from Cayman Chemicals, ascorbate from Sigma (St. Louis, MO, USA), and CsA from Meilun Corp (Dalian, China) [35].

### 2.2. Primary Cultures of Epithelial Cells of Chick Embryo Cecum and Parasite Infections

In accordance with previous studies [31], we collected embryonic cecal epithelial cells from 15-day-old chicken embryos. In brief, the cells were digested in 50 mg/L thermolysin (Sigma) at 41 °C for 120 min and then cultured in complete DMEM at 41 °C in 8% CO_2_. Confluent cells were infected with 3 × 10^5^ sporozoites per well in 6-well plates and 1 × 10^5^ sporozoites per well in 48-well plates of freshly excised *E. tenella* sporozoites.

### 2.3. MTT Assays

Cell viability was evaluated via 3-(4,5-Dimethylthiazol-2-yl)-2,5-diphenyltetrazolium bromide (MTT) assays following exposure to diazoxide (Sigma). Briefly, cells in 96-well plates were treated with N, N, N’, N’-tetramethyl-1,4-phenylenediamine (TMPD) (12.5–100 μM) + ascorbate (Asc) (0.2 mM) for 4, 24, and 48 h. Absorbance was measured at 492 nm, and values were normalized to untreated controls from triplicate wells.

### 2.4. Experimental Design and Grouping for Chick Embryo Cecal Epithelial Cell Infection and TUNEL Assays

Cecal epithelial cells in 6-well plates (2 × 10^5^ cells per well) were infected with 3 × 10^5^ *E. tenella* sporozoites. For terminal deoxynucleotidyl transferase-mediated dUTP nick end labeling (TUNEL) assays, 3.1 × 10^4^ cells grown on glass coverslips were infected with 1 × 10^5^ sporozoites and treated under four conditions: (1) positive controls (*E. tenella* sporozoite-infected; group T0), (2) negative controls (group C), (3) infected + cyclosporine A (CsA) (20 μM) for 24 h (group T1), and (4) infected + 50 μM TMPD + 0.2 mM Asc for 4 h (group T2). All experiments were performed at least three times, with five wells per group in each replicate.

### 2.5. H&E Staining

*E. tenella* sporozoite infection was evaluated by H&E staining. At 4, 24, 48, 72, 96, and 120 h post-infection, cells in 48-well plates were fixed in Bouin’s solution and washed in 70% ethanol for 30 min. After washing in water for 15 min, the cells were stained with Lillie–Mayer’s hematoxylin (Solarbio) for 30 min. Slides were sequentially soaked in water, 1% HCl, and 70% ethanol for 2 min each, followed by dehydration using a graded ethanol series (50%, 70%, 80%, 90%, and 95%). Slides were then immersed in 1% eosin (Solarbio) for 25 min, dehydrated in absolute ethanol, cleared in xylene, and finally mounted with neutral balsam. The infection rates of *E. tenella* were determined by examining 200 randomly selected fields of view.

### 2.6. Annexin/PI Staining

Externalized phosphatidylserine served as a marker of apoptotic cells, detected by a commercial Annexin V/Dead Cell Apoptosis kit (Thermo Fisher Scientific, Waltham, MA, USA). Viable cells remained unstained, whereas early apoptotic cells were Annexin V-positive, and late apoptotic or necrotic cells were positive for both Annexin V and PI. Prior to staining, the cells were trypsinized, washed in PBS, and infected. The cells were incubated in a binding buffer for 30 min and stained with Annexin V/PI. Flow cytometric analysis was performed on a FACSCalibur (BD Biosciences, San Jose, CA, USA) equipped with an argon laser (488 nm). Cells were stained with Annexin V and propidium iodide (PI) to assess apoptotic stages. Forward scatter (FSC) and side scatter (SSC) parameters were used to distinguish between viable, early apoptotic, and late apoptotic or necrotic cells. Data acquisition was performed using a logarithmic scale for fluorescence intensity, collecting at least 10,000 events per sample. Flow cytometric data were analyzed with CellQuest Pro 5.1 software (BD Biosciences, Milpitas, CA, USA), and the apoptotic cell population was gated according to the Annexin V and PI staining patterns.

### 2.7. Cytochrome c Assays

We isolated the mitochondrial fraction using a commercial mitochondria isolation kit (Beyotime, Shanghai, China). After centrifugation, the supernatants were examined for cytochrome c content by ELISA (Atibodies-online, Aachen, Germany). Absorbance was measured at 450 nm, with background values subtracted at 540 nm. Cytochrome c concentrations were calculated from the standard curve. To ensure the accuracy and reproducibility of the measurements, all samples and standards were analyzed in duplicate measurements. A blank control was included to eliminate background interference. The assay was performed under standardized conditions, which included consistent incubation times and temperature regulation, to minimize experimental variability. If the absorbance of a sample exceeded the highest standard, the sample was diluted as needed and reanalyzed to obtain an accurate quantification.

### 2.8. Caspase-9 Activity

Caspase-9 activity was measured using a commercial kit (Beyotime). At the indicated time points post-infection, the cells were trypsinized and resuspended in cell lysis buffer, and 10 μL of the specific caspase-9 substrate (Ac-DEVD-pNA or Ac-LEHD-pNA) was added to each sample. The absorbance of the released pNA was measured at 405 nm to quantify caspase-9 activity [36,37].

### 2.9. Statistics

The data were compared via a one-way ANOVA using SPSS 22.0 software. They are presented as the mean ± SD. Significant *p*-values were less than 0.05.

## 3. Results

### 3.1. Apoptosis Assessments in Response to Parasite Infection

**Figure 1:**
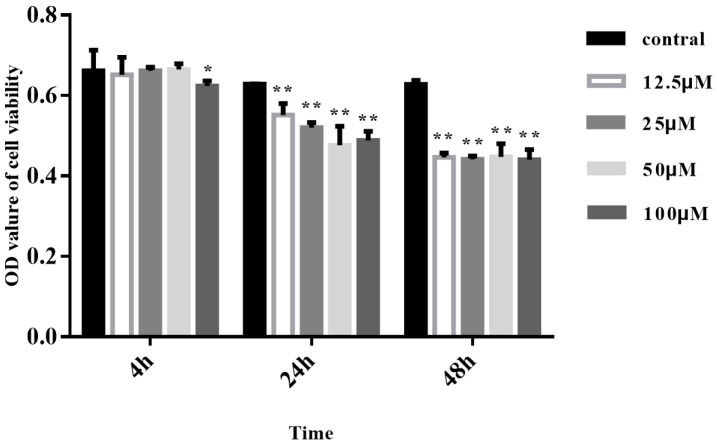
MTT assays as per the mentioned treatment methods. Controls vs. * *p* < 0.05, ** *p* < 0.01.

We first examined the effects of TMPD + Asc on cell viability using MTT assays. As shown in Figure 1, at 4 h, cells treated with TMPD + Asc showed no significant loss of viability (*p* > 0.05). However, apoptotic rates increased significantly at later time points and higher concentrations (≥48 h) (*p* < 0.05). Based on these findings, cells were incubated with 50 μM TMPD + 0.2 mM Asc for 4 h prior to subsequent assays (Figure 1).

### 3.2. Effect of CsA, TMPD, and Asc on the Infection Rates of E. tenella

**Figure 2:**
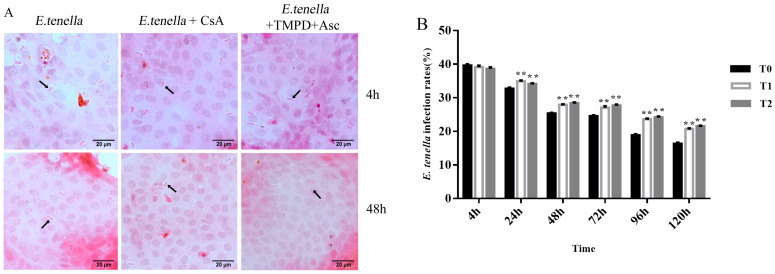
(**A**) Rates of *E. tenella* infection assessed by H&E staining. Arrows: sporozoites. (**B**) Quantification of A (n = 5). Data are presented at the mean ± SE. ** *p* < 0.01 vs. T0.

At 4 h post-infection, no significant differences were observed in *E. tenella* sporozoite infection levels between the CsA- or TMPD + Asc-treated groups and the untreated group (*p* > 0.05) (Figure 2B). However, from 1 to 5 days post-infection, cells treated with TMPD + Asc or CsA demonstrated a significant increase in *E. tenella* infection levels compared to the untreated group (*p* < 0.01). The infection rates in the CsA and TMPD + Asc treatment groups increased progressively compared to the untreated group between 24 and 72 h, reaching a peak at 96–120 h. Although the overall infection rate declined at later stages, the increasing trend in infection levels over time and the distinct effects of TMPD + Asc and CsA treatments, both resulting in significantly elevated infection levels relative to the untreated group (*p* < 0.01), were evident in the graphical representation of data (Figure 2A,B).

### 3.3. Inhibiting Cytochrome c Reduces E. tenella-Induced Apoptosis

**Figure 3:**
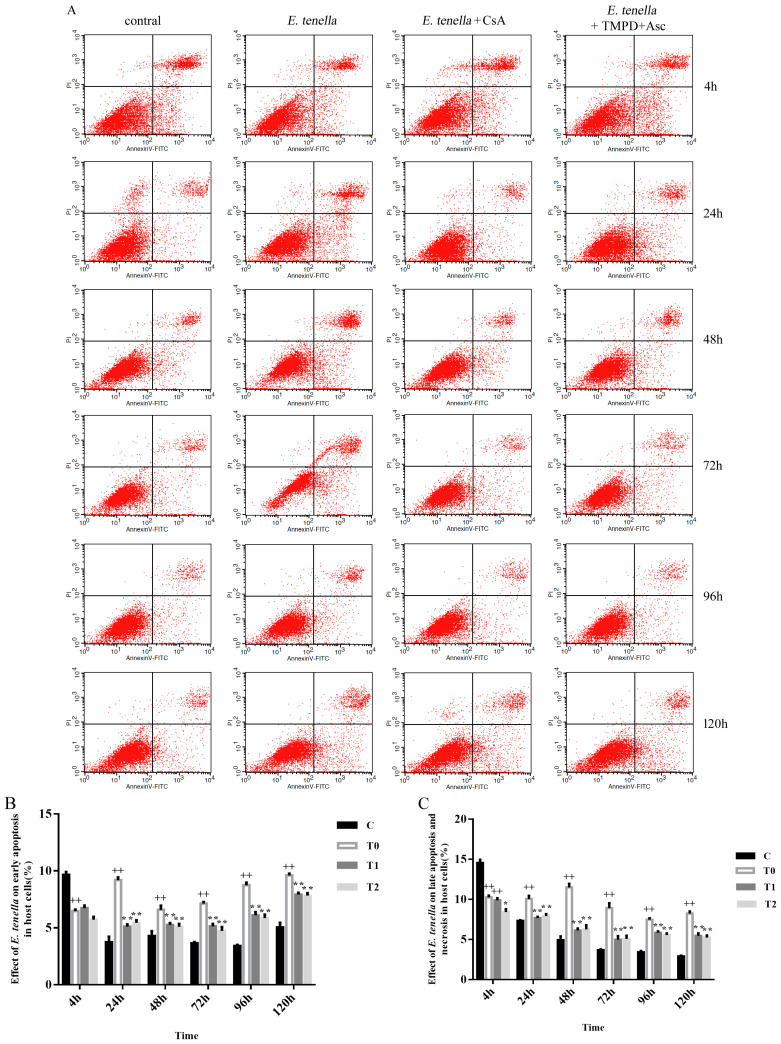
(**A**) Annexin/PI staining following infection. Quadrants in lower left: viable cells (Annexin V−/PI−); lower right quadrant: cells in early apoptotic stage (annexin V+/PI−); quadrant in upper right: cells undergoing late apoptosis and/or necrosis (annexin V+/PI+) (**B**) Measurement of early apoptosis (n = 5). (**C**) Measurement of late necrosis/apoptosis (n = 5). Note: vs. group C, ++ *p* < 0.01; vs. group T0, * *p* < 0.05, ** *p* < 0.01.

At 4 h post-infection, the apoptosis rate in sporozoite-infected cells (T0) was lower than that in uninfected controls (group C) (*p* < 0.01), suggesting that *Eimeria tenella* may transiently suppress host cell apoptosis at the early stage of infection. From 1 to 5 days post-infection, early apoptotic rates of *E. tenella*-infected cells were higher in T0 than in group C (*p* < 0.01). This trend is clearly illustrated in Figure 3A, where the fluorescence intensity in the lower right quadrant (Annexin V+/PI−) is significantly enhanced in the T0 group relative to group C, suggesting a higher proportion of early apoptotic cells in the infected group. In contrast, from 24 to 120 h post-infection, early apoptotic rates in CsA-treated (T1) and TMPD + Asc (T2) groups were significantly lower than in T0 (*p* < 0.01). No differences in apoptosis were observed between T1 and T2 (*p* > 0.05) (Figure 3A,B). This is also evident in Figure 3A, where the early apoptotic quadrant in T1 and T2 exhibits notably reduced fluorescence intensity compared to T0.

At 4 h post-infection, late apoptosis/necrosis was less frequent in T0 than in group C (*p* < 0.01). By contrast, from 24 to 120 h post-infection, T0 showed higher apoptotic rates than group C (*p* < 0.01). At 4 h post-infection, T1 and T0 exhibited comparable apoptosis rates, whereas T2 displayed lower apoptosis rates than T0 (*p* < 0.05). From 24 to 120 h post-infection, the T1 and T2 groups exhibited significantly lower apoptosis rates than T0 (*p* < 0.01), indicating that both CsA and TMPD + Asc effectively mitigated *E. tenella*-induced apoptosis at later time points. This trend is also reflected in Figure 3A, where the fluorescence intensity in the upper right quadrant (Annexin V+/PI+) is markedly increased in T0 compared to group C, while T1 and T2 demonstrate reduced fluorescence intensity in this quadrant, further confirming their inhibitory effects on late apoptosis and necrosis. No significant differences were observed between the T1 and T2 groups (*p* > 0.05) (Figure 3A,C).

### 3.4. Cytochrome c Activity

**Figure 4:**
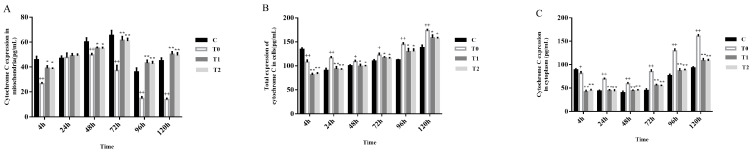
Densitometric analysis of cytochrome c expression (n = 5). (**A**) Mitochondria. (**B**) Total cell. (**C**) Cytoplasm. Controls vs. + *p* < 0.05, ++ *p* < 0.01; T0 vs. * *p* < 0.05, ** *p* < 0.01.

From 48 to 120 h post-infection, mitochondrial cytochrome c levels in T0 gradually decreased, showing a significant difference (*p* < 0.01) compared with group C. From 72 to 120 h post-infection, mitochondrial cytochrome c expression in T1 and T2 was higher than in T0 (*p* < 0.01). No significant differences in mitochondrial cytochrome c expression were observed between T1 and T2 at any time point (*p* > 0.05) (Figure 4A).

At 4 h post-infection, total cytochrome c expression was significantly lower in T0 than in group C (*p* < 0.01). From 24 to 120 h post-infection, cytochrome c levels in T0 gradually increased and were significantly higher than in group C (*p* < 0.01 or *p* < 0.05). Although cytochrome c expression in T1 and T2 also increased from 4 to 120 h post-infection, it remained lower than in T0, and the differences were not statistically significant (Figure 4B).

At 4 h post-infection, cytoplasmic cytochrome c levels were lower in T0 than in group C (*p* < 0.05). From 24 to 120 h post-infection, cytoplasmic cytochrome c in T0 host cells gradually increased, reaching significantly higher levels than in group C (*p* < 0.01). Cytochrome c levels in T1 and T2 increased from 4 to 120 h post-infection but remained considerably lower than in T0 (Figure 4C).

Together, cytoplasmic cytochrome c levels also increased in T0 compared with group C (Figure 4). Simultaneously, its mitochondrial distribution decreased (Figure 4A). However, no significant differences were found between T1 and T2.

### 3.5. Relationship Between Cytochrome c and Caspase-9 Activity Following E. tenella Infection

**Figure 5:**
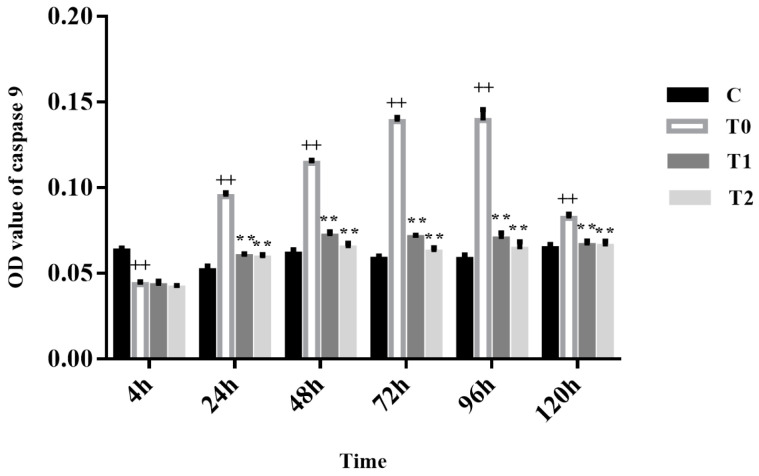
Measurement of caspase-9 activity. ++ *p* < 0.01 vs. group C; ** *p* < 0.01 vs. T0.

After 4 h of infection, T0 showed lower levels of caspase-9 activity compared to group C (*p* < 0.01). At 24 to 120 h after infection, T0 cells displayed increased caspase-9 activity compared to group C (*p* < 0.01). At 24 to 120 h post-infection, both T1 and T2 cells had reduced levels of caspase-9 activity vs. T0 cells (*p* < 0.01), with no significant difference between T1 and T2 (*p* > 0.05). Caspase-9 activity gradually decreased throughout the infection (Figure 5).

## 4. Discussion

Mitochondria-mediated apoptosis occurs in intestinal epithelial cells following *E. tenella* infection [31], though the underlying molecular mechanisms are not fully elucidated. Cytochrome c is a key apoptotic inducer, but its connection to *E. tenella* infection remains unclear. This study investigated cytochrome c-mediated mitochondrial apoptosis and its role in *E. tenella* infection of in vitro cecal epithelial cells.

We first examined whether TMPD + Asc affects parasite infection. TMPD and Asc can alter the redox state of cytochrome c [38,39,40] and have been shown to exhibit cell permeability under specific conditions [41,42,43]. Borutaite et al. [44] demonstrated that TMPD and Asc exhibit anti-apoptotic properties. The MPTP is a pore situated on the mitochondrial inner membrane, whose excessive opening can cause mitochondrial dysfunction and initiate apoptotic pathways [45,46]. CsA inhibits the MPTP and likewise protects cells from apoptosis [47,48].

Caspase-9 is a key initiator caspase in the intrinsic apoptosis pathway. It is activated by mitochondrial dysfunction, where cytochrome c is released from the mitochondria into the cytoplasm, which then forms the apoptosome with Apaf-1. This complex activates caspase-9, which in turn activates downstream effector caspases, such as caspase-3 and caspase-7, ultimately leading to the execution of apoptosis [49,50]. At 4 h post-infection, cells in late or early apoptotic stages and caspase-9 activity were reduced in infected cells compared with uninfected cells. CsA and TMPD + Asc did not affect parasite infection at this early stage. These findings suggest that *E. tenella* maintains host cell viability at early stages (4 h post-infection), thereby permitting the completion of its parasitic lifecycle [21,22,51]. Compared to the apoptosis mechanism in *Eimeria tenella* infection, *Plasmodium* also promotes their survival by regulating host cell apoptosis [52]. *Plasmodium* activates host cell anti-apoptotic pathways (such as Bcl-2 family proteins), delaying red blood cell death, thus creating a more favorable environment for the parasite’s survival. However, at later time points (24, 48, 72, 96, and 120 h post-infection), CsA and TMPD + Asc significantly reduced the number of apoptotic cells relative to the T0 group (*p* < 0.01). In *E. tenella* infection of primary epithelial cell cultures, the apoptotic response, including cytochrome c release and caspase-9 activation, peaks at 96 to 120 h post-infection, which coincides with the rupture of second-generation schizonts and the onset of hemorrhagic lesions in vivo.

Cytochrome c release is a prerequisite for caspase activation [53]. The mitochondrial permeability transition pore (MPTP) regulates the mitochondrial membrane potential and thus mitochondrial activity [54,55,56]. We quantified cytochrome c release following *E. tenella* infection by ELISA. The CsA group exhibited higher levels of mitochondrial cytochrome c than the T0 group from 4 to 120 h post-infection, whereas cytoplasmic cytochrome c was lower in CsA-treated cells. Cytoplasmic cytochrome c increased throughout infection, consistent with apoptotic induction from 24 to 120 h, as reported previously [57]. A similar pattern was observed in the TMPD + Asc group. Moreover, both CsA and TMPD significantly reduced apoptosis. These findings underscore the roles of the MPTP and cytochrome c as central integrators of the host apoptotic response to parasite infection [22,58].

The MPTP opens to prevent excessive cation accumulation in the mitochondrial intermembrane space [54,59,60]. Upon MPTP opening, cytochrome c is released, interacting with Apaf-1, dATP, and procaspase-9. Caspase-9 is then activated, leading to apoptosis [61,62,63]. Our results suggest the release of mitochondrial cytochrome c into the cytoplasm following *E. tenella* infection triggers caspase-9 activation in infected cells. These observations align with Li et al., who demonstrated that *E. tenella* activates caspase-9 and caspase-3 via the intrinsic apoptotic pathway.

TMPD modulates the redox state to reduce oxidative stress, indirectly suppressing apoptosis. Cyclosporine A inhibits the opening of the MPTP, reduces intracellular calcium accumulation, and prevents the release of cytochrome c, thereby effectively suppressing intrinsic apoptosis and protecting host immune function. We show that TMPD and CsA, respectively, inhibit cytochrome c activation and MPTP opening, significantly reducing host cell apoptosis caused by *E. tenella* infection. These results indicate that mitochondrial dysfunction underlies host cell apoptosis during the late stages of *E. tenella* infection. Future work should focus on developing new strategies to inhibit *E. tenella*-induced apoptosis. Inhibition reduces the pathological effects of the infection, such as hemorrhage and tissue damage, after infection has occurred.

Inhibiting host cell apoptosis helps prolong cell survival and preserve normal immune function, thereby enhancing the immune system’s defense against *E. tenella*. Additionally, inhibiting apoptosis can alleviate clinical symptoms caused by the infection, such as diarrhea, weight loss, and intestinal damage, thereby improving poultry survival rates and production performance. At the same time, by suppressing host cell death, the immune evasion ability of *E. tenella* is reduced, thereby enhancing the host’s immune clearance capability. In addition to TMPD and CsA, other research has investigated alternative apoptosis inhibitors, such as Bcl-2 family protein modulators and natural products (e.g., quercetin, artemisinin) [64,65]. Bcl-2 modulators can more precisely regulate the apoptosis process, but they may necessitate more precise dosage control and could have potential side effects on the immune system. Natural products generally have higher safety and are suitable for long-term use, but their effects may be slower and require higher doses to show significant results.

In conclusion, parasites [29,52], including *E. tenella*, play a crucial role in modulating host cell death. By modulating apoptosis pathways, parasites provide the necessary support for the completion of their lifecycle. Understanding how *E. tenella* regulates host cell death provides potential therapeutic opportunities. Future research should prioritize the development of interventions targeting these regulatory pathways, especially during the later stages of infection. By intervening in the host cell apoptosis process, it may be possible to alleviate the pathological effects caused by parasites, such as tissue damage and immune evasion, therefore enhancing host survival and immune function. Ultimately, developing effective treatments to regulate the host’s apoptotic response will provide new therapeutic approaches for controlling parasitic infections and improving poultry health.

## 5. Conclusions

In conclusion, our findings demonstrate that *Eimeria tenella*-induced apoptosis in chick embryo cecal epithelial cells occurs via mitochondrial pathways characterized by cytochrome c release and MPTP opening. The inhibition of these processes by CsA and TMPD significantly mitigates apoptosis, indicating potential therapeutic approaches to manage *E. tenella* infections in poultry. Future research should focus on exploring alternative inhibitors targeting these pathways and validating the results in live chicken models to validate their efficacy in more biologically relevant models. This will help develop more practical therapeutic approaches to effectively control *E. tenella* infections and improve poultry health.

## Data Availability

The data presented in this study are available on request from the corresponding author.
